# Supplementary material for: The microbiome biomarkers of pregnant women’s vaginal area predict preterm prelabor rupture in Western China
Source: Front Cell Infect Microbiol. 2024 Oct 31;14:1471027. doi: 10.3389/fcimb.2024.1471027 (PMC11560878; doi:10.3389/fcimb.2024.1471027)
Supplement: Supplementary file 1 [file DataSheet1.zip › compare_1/Community/Pieplot/genus/sample/P28.genus.pieplot.pdf]

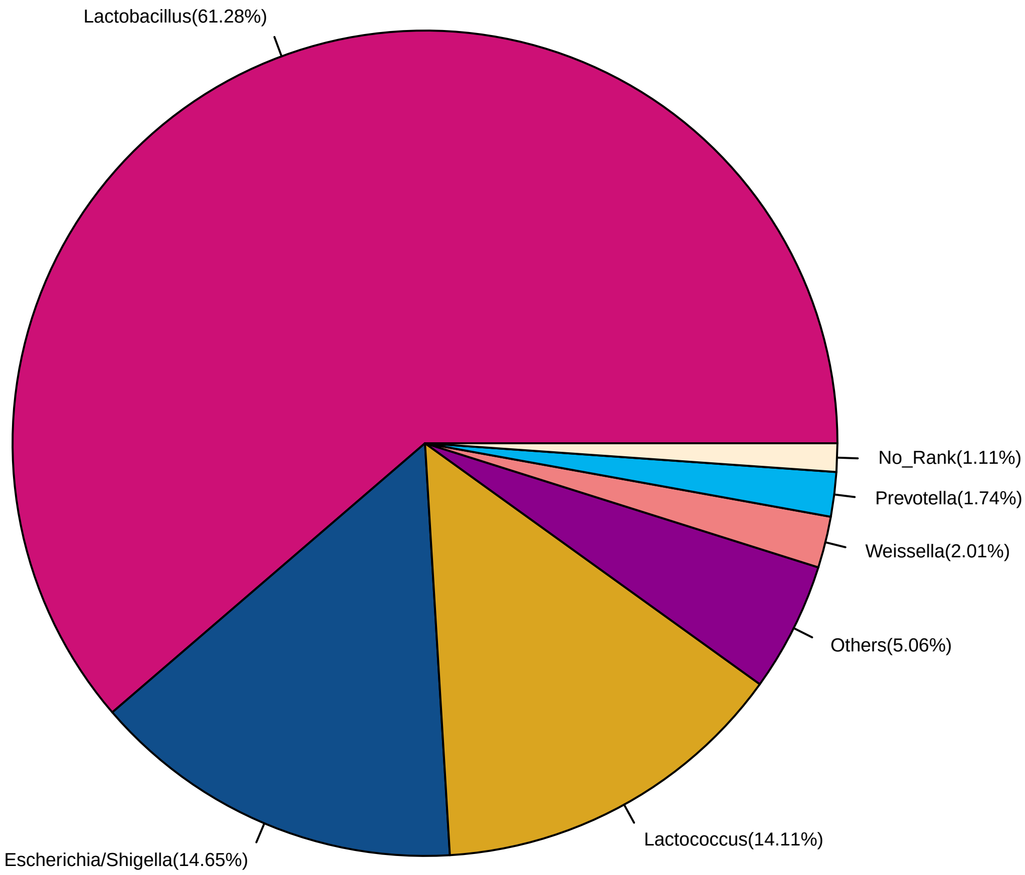
Lactobacillus(61.28%)
No_Rank(1.11%)
Prevotella(1.74%)
Weissella(2.01%)
Others(5.06%)
Lactococcus(14.11%)
Escherichia/Shigella(14.65%)
